# Non-small cell lung carcinoma presenting as carcinomatous meningitis

**DOI:** 10.4103/0970-2113.68324

**Published:** 2010

**Authors:** A. R. Paramez, Ramakant Dixit, Neeraj Gupta, Rakesh Gupta, Manoj Arya

**Affiliations:** *Department of Respiratory Medicine and Tuberculosis, J. L. N. Medical College, Ajmer, India*

**Keywords:** Adenocarcinoma lung, carcinomatous meningitis, malignant metastasis

## Abstract

Meningeal carcinomatosis is a diffuse infiltration of leptomeninges and sub arachnoid space by malignant cells metastasizing from systemic cancer. Primary bronchogenic carcinoma presenting as carcinomatous meningitis is a very rare occurrence in clinical practice, often occurring during the treatment course of the underlying malignancy. We present this rare presentation in a young non-smoker male.

## INTRODUCTION

Widespread dissemination of tumor cells throughout meninges and ventricles has been the pattern in about 5% of cases of adenocarcinoma of breast, lungs, gastrointestinal tract, melanoma, childhood leukemia and systemic lymphoma.[1] With certain carcinomas, it may be the first manifestation of a neoplastic illness,[2] although more often the primary tumor has been present and is under treatment. Meningeal carcinomatosis without brain parenchymal involvement is a rare metastatic manifestation of lung cancer. The present report describes such a case.

## CASE REPORT

A 30-year-old male non-smoker patient presented with three months of fever, pain in right sacro iliac joint and generalized weakness. The fever was high grade to start with, associated with chills and rigors. The severity of fever lowered with symptomatic treatment. Thereafter he had low grade fever off and on. The pain in the right sacroiliac region was severe, but intermittent, sometimes radiating to lower limb and partially relieved by analgesics. There was no history of burning micturition, upper or lower respiratory tract symptoms. Patient was conscious, cooperative, averagely built and poorly nourished. Pallor and oral thrush was present. Neck rigidity was present with positive kernigs sign. There was no clubbing, cyanosis, jaundice, pedal edema or generalized lymphadenopathy etc. Liver and spleen were just palpable.

**Figure 1:**
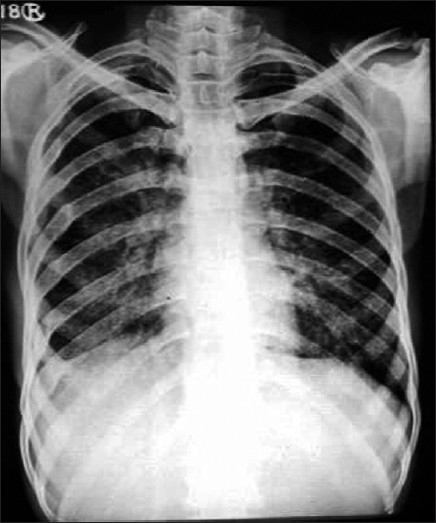
Chest radiograph showing bilateral mid and lower zone miliary nodular opacities

He was initially evaluated in a community health center, where his chest skiagram showed bilateral miliary nodular opacities in both lungs, more in mid and lower zones [Figure 1]. His sputum for acid fast bacilli was negative. Hemoglobin was 12 g/ dl with total leukocyte count 9800/mm^3^ (polymorphs 76%, lymphocytes 15%, eosinophills 7%) and ESR 55 mm in first hour. Renal and liver function tests were normal. He received antituberculosis treatment with isoniazid, rifampicin, ethambutol and pyrazinamide based on these reports, without any improvement.

On admission, his blood counts and biochemistry were normal with normal peripheral blood film. Blood was also negative for malarial parasite, Widal test, HBsAg, HIV and ANA etc. CECT head revealed slightly prominent bilateral temporal horns of lateral ventricles. His CSF analysis revealed an ADA of 7 µ/L, proteins 45.9 mg/dl, sugar 30 mg/l, AFB negative, India -Ink stain negative, sterile on pyogenic culture and a cell count of 6 cells/mm^3^. The cells were described as hypo cellular smear showing only a few isolated singly placed very large abnormal cells, having large vesicular nuclei and variable amount of cytoplasm and some were binucleated. His USG abdomen showed mild hepatosplenomegaly.

**Figure 2:**
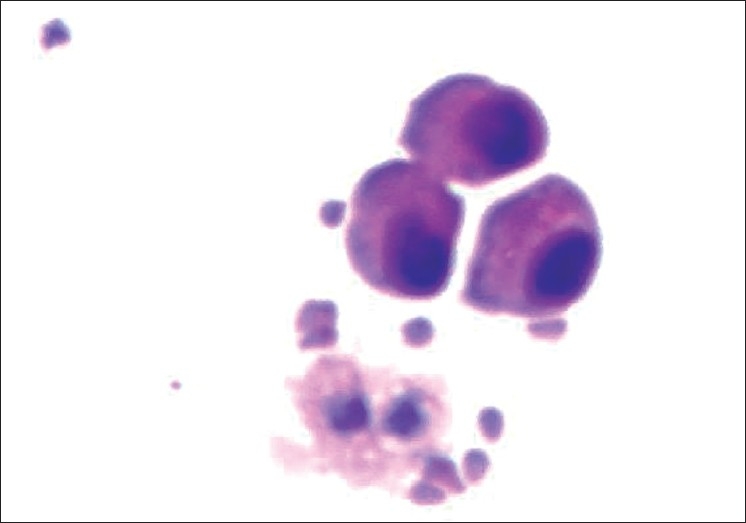
CSF smear showing abnormal atypical cells with hyper chromatic nuclei and pinkish cytoplasm (H and E, ×400)

Patient was put on intravenous mannitol and dexamethasone, switched over to oral prednisolone 40 mg and glycerol along with other supportive therapy. He had partial relief of headache and fever. After 10 days, patient again had severe headache and fever with persistent pain in sacroiliac joint. A repeat CSF analysis was done which showed normal biochemistry, but microscopy still showed very large abnormal and atypical cells having large vesicular nuclei and variable amount of cytoplasm [Figure 2]. Persistence of abnormal cells raised the doubt of malignant process. His CECT chest was done which revealed bilateral ground glass haziness in mid and lower zones of lungs with minimal right pleural effusion without any mediastinal lymphadenopathy [Figure 3a and b]. Fiberoptic bronchoscopy was performed to further evaluate the pulmonary lesion. Bronchoalveolar lavage and transbronchial biopsy was taken. Pleural fluid was aspirated. All the three specimens were positive for malignant cells- adenocarcinoma type [Figure 4].

**Figure 3a:**
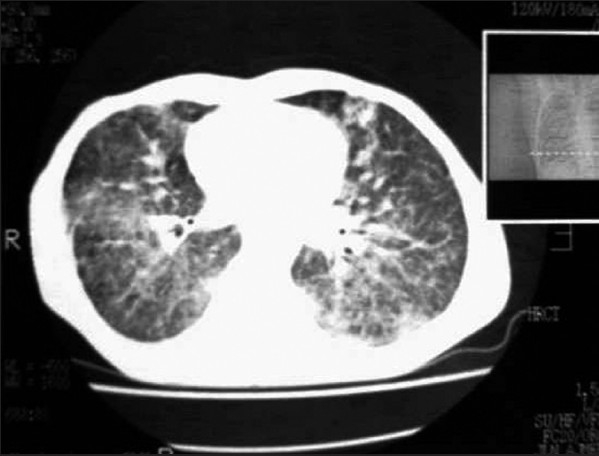
CECT chest showing bilateral micro nodular opacities in mid and lower zones

**Figure 3b:**
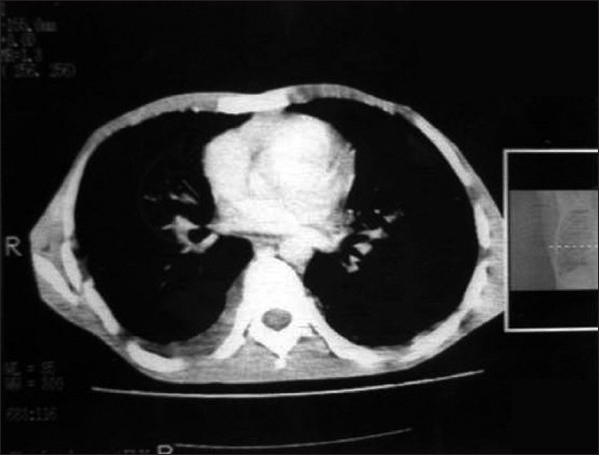
CECT chest showing mild pleural effusion on right side

**Figure 4:**
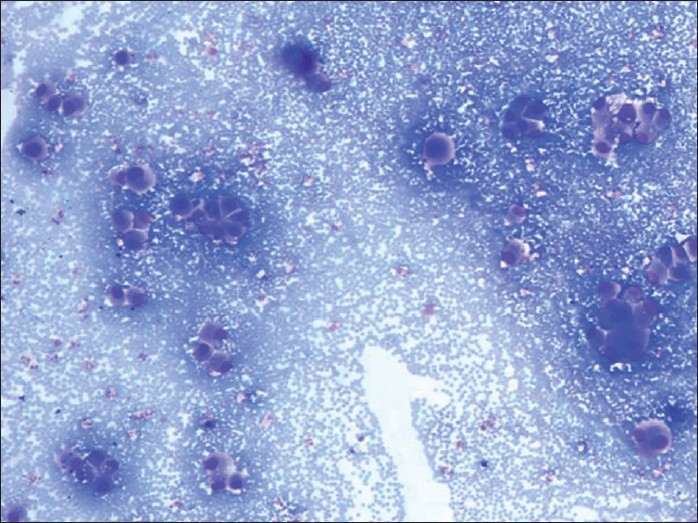
Bronchial washing smear showing very large binucleated abnormal cells with large vesicular nuclei and variable amount of cytoplasm (H and E, ×400)

The patient was immediately put on cisplatin based chemotherapy and was advised bone scan, but he expired after three days of chemotherapy.

## DISCUSSION

Carcinomatous meningitis/ meningeal carcinomatosis is a special form of metastatic cancer in which widespread dissemination of tumor cells occurs throughout the meninges and ventricles. This has been noted in almost 5% of patients with solid tumors.[2] The most commonly encountered metastatic neoplasm in CSF is adenocarcinoma and most frequent site of origin are the lung, breast, stomach and skin.[3] In lung carcinoma, meningeal carcinomatosis is far more common in small cell histology,[4]. Reported literature of carcinomatous meningitis among non- small cell carcinoma being very few[5,6] and mostly observed during the treatment course of the primary lung cancer. Our case is unique one in view of meningeal carcinomatosis as the presentation of adenocarcinoma lung.

Neoplastic meningitis is characterized by multifocal neurologic signs and symptoms. Headache and backache, often with sciatica, are common but not invariable. Polyradiculopathies, multiple cranial nerve palsies and a confusional state have been the principal manifestations. Only a small number have an uncomplicated meningeal syndrome of headache, nausea, and meningismus. Delirium, stupor, and coma follow in pure meningeal syndrome. Focal neurological signs and seizures may be associated, and may have hydrocephalus in half of patients. The evolution of all these syndromes is generally sub acute over weeks with a more rapid phase as the illness progresses.[1] Our case also presented with neurological features.

The diagnosis can be established in most cases by identifying tumor cells in the CSF using flow cytometry, cytospin, centrifugation, or Millipore filtering. More than one examination, with generous amount of CSF, may be needed. Increased pressure, elevation of protein and low glucose levels and lymphocytic pleocytosis are other common CSF findings. In some patients CSF remains persistently normal. Measuring the CSF for certain biological markers of cancer - such as lactate dehydrogenase, β glucuronidase, β_2_ microglobulin and carcinoembryonic antigen (CEA) – offers another means of making a diagnosis and following the response to therapy.[1] Other useful tests to establish diagnosis and guide treatment include magnetic resonance imaging of the brain and spine and radioisotope CSF flow studies.

Treatment consists of radiation therapy to symptomatic areas like cranium, spine, and posterior fossa, followed in selected cases by the intraventricular administration of methotrexate at a dose of 10 – 15mg either daily for three to four days or at three-day intervals for 15 days. The intrathecal chemotherapy has also been tried with cytarabine, thiotepa and gemcitabine.[7] Involvement of cranial nerves or an encephalopathy due to widespread infiltration of cranial meninges is treated with whole- brain radiation, 300 cGy for 10 days.[1]

CSF metastasis correlates with worse prognosis and patient survival. The reported median duration of survival after diagnosis is four to six weeks if left untreated and two to three months if treated. Best response to treatment occurs in patients with lymphomas, breast cancer and small cell lung cancers; prognosis in other lung cancers, melanomas and adenocarcinomas is bleak.[8] Early recognition of neoplastic cells in CSF can be useful in view of early treatment that may contribute to quality of life, control of neurological symptoms and longer survival.
